# Case Report: Differential Diagnosis of Lower Extremity Weakness in a Young Male - Consider Foix Alajouanine Syndrome

**DOI:** 10.5811/cpcem.2021.8.52660

**Published:** 2022-01-04

**Authors:** Avi Siani, Alexander Garrett, Natasha Thomas

**Affiliations:** Harbor-UCLA Medical Center, Department of Emergency Medicine, Torrance, California

**Keywords:** case report, Foix-Alajouanine, spinal arteriovenous malformations, congestive myelopathy

## Abstract

**Introduction:**

There is a limited list of emergent spinal cord pathology that must be considered in patients with focal neurological deficits in the emergency department. Identification of these conditions requires a detailed history and neurological exam and may also require advanced testing and imaging.

**Case Report:**

Here we present the case of a patient with a rare arteriovenous malformation of the spinal cord vessels causing congestive myelopathy (Foix-Alajouanine syndrome) that presented as a clinical mimic of spinal cord compression.

**Conclusion:**

Emergency physicians should be aware of Foix-Alajouanine syndrome, as its workup and management differ from more common pathologies that may present similarly.

## INTRODUCTION

Acute or progressive onset of bilateral lower extremity weakness, sensory loss, hypo- or hyperreflexia, and bladder dysfunction are concerning for emergent spinal pathology. These may include cauda equina syndrome, transverse myelitis, spinal cord compression, or spinal trauma. Demyelinating diseases such as Guillain-Barré syndrome (GBS), as well as chronic inflammatory demyelinating polyneuropathy (CIDP) must also be considered in the emergency department (ED).^1^ Foix-Alajouanine syndrome is a rare disease described as intradural arteriovenous malformations (AVM) that can cause venous congestion and ischemic myelopathy, ultimately leading to a clinical presentation similar to the above diagnoses.^2^ The syndrome was initially described by Foix and Alajouanine in 1926 as acute or subacute neurological deterioration in a patient who was found to have tortuous vessels lying on the surface of the spinal cord with associated necrosis on autopsy.^3^

In 1931 Lhermitte found an association with the syndrome and spinal AVMs.^4^ Initially, it was hypothesized that these AVMs were likely to become thrombosed, resulting in ischemic and necrotic myelopathy. Based on this understanding of the syndrome, treatment and reversal of the progressive myelopathy was considered futile. However, recent literature shows that spinal venous thrombosis may not be responsible for the myelopathy. Rather, dural arteriovenous fistulas may cause congestive myelopathy from venous hypertension.^5^,^6^ In this scenario, endovascular repair of the fistulas is likely to improve the likelihood of a meaningful neurological recovery.

Diagnosing the condition requires a high level of suspicion as the necessary diagnostic modalities are not typically included in the ED workup for other emergent spinal pathologies. To the best of our knowledge, the ED workup and management for this rare syndrome has not been reviewed in the literature. Here we describe the case of a patient who presented to the ED with Foix-Alajouanine syndrome, with an emphasis on in-department workup and management.

## CASE REPORT

A 38-year-old male presented to the ED with progressive bilateral lower extremity weakness and difficulty with ambulation for three months, acute urinary retention since waking up that morning, and lower back pain. He denied any saddle anesthesia, leg numbness, bowel incontinence, weight loss, intravenous drug use, fevers, or recent trauma. Of note, the patient had been admitted one month prior to presentation with septic shock secondary to cholangitis and pancreatitis. He had noted bilateral lower extremity weakness at the time, but this was thought to be secondary to deconditioning from the prolonged admission. The patient otherwise had a history of schizophrenia and hypothyroidism, with no prior surgical history.

**Image 1:**
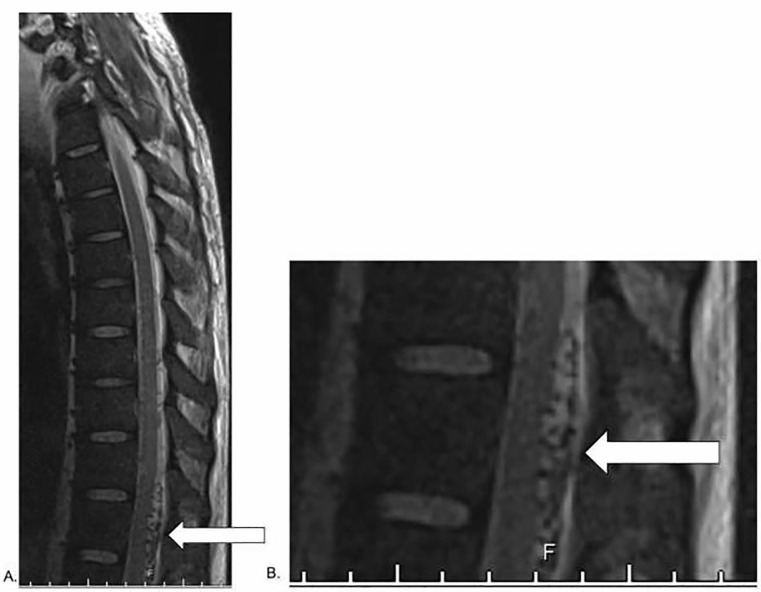
A) T2-weighted magnetic resonance imaging of the thoracic spine without contrast showing flow voids (white arrow) in the intradural extramedullary region posterior to the spinal cord from the 11th thoracic vertebra to the first lumbar vertebra consistent with spinal arteriovenous malformation. B) Close-up image of flow void (white arrow).

On exam, he had 2/5 motor strength in the bilateral lower extremities with hip flexion and extension and knee flexion and extension, and 3/5 strength with ankle plantar flexion and dorsiflexion. He was unable to safely stand or walk without assistance. His sensation was intact to light touch throughout. Achilles and plantar reflexes were absent bilaterally, with a normal Babinski sign. Rectal tone was not checked due to patient preference. The patient had a distended bladder, and one liter of urine was drained immediately following Foley catheter placement, confirming concern for acute urinary retention. Given the concern for CIDP, cauda equina syndrome, and GBS, an immediate thoracic and lumbar spine magnetic resonance imaging (MRI) was ordered and neurology and neurosurgery were consulted. Although the MRI was initially interpreted as showing no acute pathology, further review of imaging with radiology was concerning for a vascular lesion along the lumbar spine (Image 1).

**CPC-EM Capsule**

What do we already know about this clinical entity?

*Foix-Alajouanine syndrome is a rare congestive myelopathy from spinal cord arteriovenous malformations that presents as acute or subacute neurological deterioration*.

What makes this presentation of disease reportable?

*The patient in our case presented with symptoms that were a near mimic of other emergent spinal cord pathologies*.

What is the major learning point?

*This syndrome can mimic other spinal cord pathologies; it can present as flow voids on MRI, which should prompt spinal angiography to confirm the diagnosis*.

How might this improve emergency medicine practice?

*Being aware of this syndrome could expedite diagnosis, as well as minimize risks of poor outcomes from contraindicated lumbar punctures*.

**Image 2:**
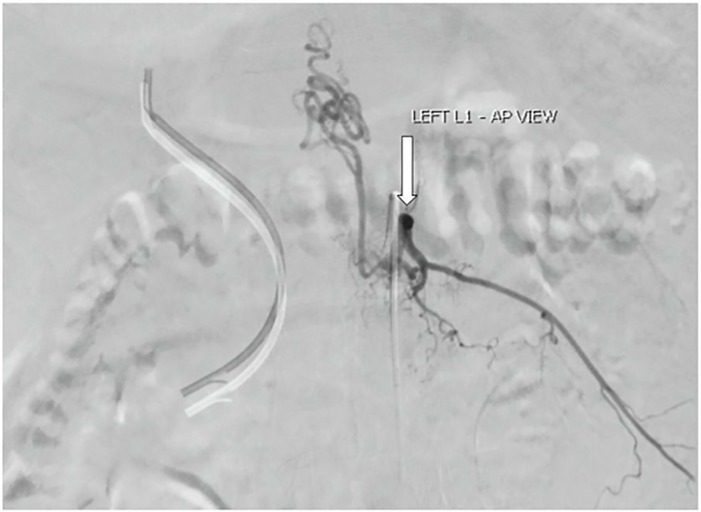
Spinal angiogram showing dural arteriovenous fistula (white arrow) arising from left lumbar artery of the first lumbar vertebra (L1) between a dural branch of the spinal ramus of a radicular artery and an intradural medullary vein. Also shown is the venous component coming off the arteriovenous malformation, which is coiled and extends superiorly to the sixth thoracic vertebra (T6) level and inferiorly to the fifth lumbar vertebra (L5) level. *AP*, anteroposterior.

Neurosurgery noted multiple flow voids from the 11th thoracic (T11) to the first lumbar (L1) vertebra on MRI, which was consistent with spinal arteriovenous fistula and Foix-Alajouanine syndrome. Neurosurgery and neurology recommended that the patient undergo spinal angiography to better characterize the vascular lesion. Additionally, both recommended that interventional radiology (IR) be consulted for an IR-guided lumbar puncture (LP) as GBS and CIDP were both still on the differential. Interventional radiology-guided LP was advised to minimize the risk of affecting the AVM. Spinal angiography showed a type one dural arteriovenous fistula supplied by a radiculomedullary branch that arose from the left L1 lumbar artery (Image 2), with a coiled venous component extending superiorly to the T6 level and inferiorly to the L5 level.

A nerve conduction study showed no evidence of demyelination. A repeat MRI of the spinal cord confirmed flow voids in the intradural extramedullary region posterior to the spinal cord from T11 to L1 consistent with spinal AVM. The patient was subsequently taken to surgery with IR placement of a 3-millimeter coil into the L1 feeder vessel for intraoperative identification, followed by a T12 through L2 laminectomy in addition to ligation of the dural arteriovenous fistula, which was performed by neurosurgery. The patient had a prolonged postoperative course complicated by wound dehiscence and poor nutrition requiring a lengthy stay at a rehabilitation facility. Approximately two months postop, the patient was able to ambulate with a walker, and was discharged home from the rehab facility. He had a follow-up MRI at six months that showed resolution of the previously identified flow voids. Eight months after the initial presentation, the patient was able to ambulate safely with a walker, had no incontinence or retention symptoms, and had 4/5 strength in his bilateral lower extremities.

## DISCUSSION

Back pain with associated bowel and/or bladder incontinence, saddle anesthesia, numbness, or weakness of the lower extremities is concerning for emergent spinal pathology. These symptoms should prompt a differential diagnosis including cauda equina syndrome, epidural hematoma, epidural abscess, cord compression or injury from trauma, transverse myelitis, CIPD, and GBS. Foix-Alajouanine syndrome is not considered in most emergency clinicians’ differential diagnosis for emergent spinal cord pathology; however, the management and diagnosis of the disease are unique enough to warrant understanding of this syndrome. Foix-Alajouanine syndrome describes a non-compressive myelopathy from venous hypertension in spinal AVMs, leading to decreased perfusion of the spinal cord and eventual cord necrosis.^7^,^8^

Regardless of the location of the AVM, the lower spinal cord seems to be predominantly affected. This is likely the result of the intraspinal veins lacking valves; thus, with gravity the lower cord becomes more congested. Also, there are fewer collateral veins in the lower part of the spinal cord to reduce the amount of venous hypertension caused by the AVM.^9^ Due to this, patients often present with lower spinal cord myelopathies that involve acute or subacute lower extremity weakness and numbness, as well as urinary retention and bowel incontinence. These symptoms can be seen in cauda equina syndrome, CIDP, and GBS, which are “cannot miss” diagnoses considered commonly in the ED. Spinal cord MRI is often performed in the evaluation of these symptoms, and a LP may be considered. Unfortunately, MRI is not completely sensitive or specific for Foix-Alajouanine syndrome, and a traditional LP is contraindicated as the provider may cause vascular injury.

There have not been any studies on the sensitivity of MRI for diagnosing spinal arteriovenous lesions; however, studies have shown that it may take years to accurately diagnose these patients. One study showed that 40–63% of patients have the disease for one to three years, and 10–34% have it for more than three years before they are accurately diagnosed.^9^ The MRI of an affected patient may show swelling of the medullary conus and some central spinal cord enhancement. On sagittal T2-weighted MRI, one should look for vascular flow voids on either the dorsal or ventral aspect of the spinal cord. If suspicion is high for spinal AVM and Foix-Alajouanine syndrome, the gold standard for diagnosis is spinal angiography, which would typically follow MRI.^10^,^11^ Given the rarity of the disease, epidemiological data are limited; however, one study reported that patients are typically male and in the sixth decade of life when diagnosed with spinal dural arteriovenous fistulas.^12^ Non-IR guided LP is contraindicated in these patients due to the chance of puncturing the vascular lesion.

The management of Foix-Alajouanine syndrome or spinal AVMs should include immediate neurosurgical consultation and avoidance of steroids and blind LP. Theoretically, keeping the patient supine could be of benefit as well, as being upright can worsen spinal venous congestion due to gravity; however. this is purely speculative based on the pathophysiology and has not been formally studied. Additionally, maintaining high systemic mean arterial pressures (MAP), which is usually advised in traumatic spinal cord injuries, would be contraindicated as higher MAPs may actually worsen venous congestion. Studies have shown that steroid use in patients with spinal arteriovenous fistulas can acutely worsen motor and sensory symptoms.^13^

Ultimately, these patients are managed by either endovascular embolization of the AVM or clipping and ligation of the AVM.^14^ Foix-Alajouanine syndrome is a rare cause of spinal cord pathology that may mimic more common diseases such as cauda equina syndrome and cord compression. It should be considered in patients with lower cord symptoms, as the workup and management in the ED differs from other myelopathies and demyelinating diseases.

## CONCLUSION

Foix-Alajouanine syndrome is a rare condition involving congestive myelopathy originating from spinal arteriovenous malformations, which can present similarly to other spinal cord pathologies that are considered to be emergent. However, the workup and management of Foix-Alajouanine largely deviates from other similar pathologies; so, albeit a rare diagnosis, it is important for emergency physicians to be familiar with this disease.

## References

[1] Menon KV, Sorour TM, Raniga SB (2014). Foix-Alajouanine syndrome presenting as acute
cauda equina syndrome: a case report. Global Spine
J.

[2] Mishra R, Kaw R (2005). Foix-Alajouanine syndrome: an uncommon cause of
myelopathy from an anatomic variant circulation. South
Med
J.

[3] Foix C, Alajouanine T (1926). La myélite nécrotique
subaigue. Rev Neurol
(Paris).

[4] Lhermitte J, Friboury-Blanc A, Kyriaco N (1931). La gliose angéio-hyperthrophique de la
moelle épinière (myélite nécrotique de
Foix-Alajouanine. Rev Neurol
(Paris).

[5] Ferrell AS, Tubbs RS, Acakpo-Satchivi L (2009). Legacy and current understanding of the
often-misunderstood Foix-Alajouanine syndrome. Historical
vignette. J
Neurosurg.

[6] Krishnan P, Banerjee TK, Saha M (2013). Congestive myelopathy (Foix-Alajouanine syndrome)
due to intradural arteriovenous fistula of the filum terminale fed by
anterior spinal artery: case report and review of
literature. Ann Indian Acad
Neurol.

[7] Heros RC (2009). Foix-Alajouanine syndrome: What is
it?. J
Neurosurg.

[8] Aminoff MJ, Barnard RO, Logue V (1974). The pathophysiology of spinal vascular
malformations. J Neurol
Sci.

[9] Jellema K, Tijssen CC, van Gijn J (2006). Spinal dural arteriovenous fistulas: a congestive
myelopathy that initially mimics a peripheral nerve
disorder. Brain.

[10] Doppman JL, Di Chiro G, Dwyer AJ (1987). Magnetic resonance imaging of spinal arteriovenous
malformations. J
Neurosurg.

[11] Krings T, Mull M, Gilsbach JM (2005). Spinal vascular
malformations. Eur
Radiol.

[12] Jellema K, Canta LR, Tijssen CC (2003). Spinal dural arteriovenous fistulas: clinical
features in 80 patients. J Neurol Neurosurg
Psychiatry.

[13] Nasr DM, Brinjikji W, Rabinstein AA (2017). Clinical outcomes following corticosteroid
administration in patients with delayed diagnosis of spinal arteriovenous
fistulas. J Neurointerv
Surg.

[14] Krings T, Thron AK, Geibprasert S (2010). Endovascular management of spinal vascular
malformations. Neurosurg
Rev.

